# Development of a Flex and Stretchy Conductive Cotton Fabric Via Flat Screen Printing of PEDOT:PSS/PDMS Conductive Polymer Composite

**DOI:** 10.3390/s20061742

**Published:** 2020-03-20

**Authors:** Granch Berhe Tseghai, Benny Malengier, Kinde Anlay Fante, Abreha Bayrau Nigusse, Lieva Van Langenhove

**Affiliations:** 1 Department of Materials, Textiles and Chemical Engineering, Ghent University, 9000 Gent, Belgium; Benny.malengier@UGent.be (B.M.); AbrehaBayrau.BayrauNigusse@UGent.be (A.B.N.); Lieva.VanLangenhove@UGent.be (L.V.L.); 2 Ethiopian Institute of Textile and Fashion Technology, Bahir Dar University, 6000 Bahir Dar, Ethiopia; 3 Jimma Institute of Technology, Jimma University, Jimma, Ethiopia; kinde.anlay@ju.edu.et

**Keywords:** conductive polymer composite, PEDOT:PSS, flexible electronics, wearable application

## Abstract

In this work, we have successfully produced a conductive and stretchable knitted cotton fabric by screen printing of poly(3,4-ethylenedioxythiophene) polystyrene sulfonate (PEDOT:PSS) and poly(dimethylsiloxane-b-ethylene oxide)(PDMS-b-PEO) conductive polymer composite. It was observed that the mechanical and electrical properties highly depend on the proportion of the polymers, which opens a new window to produce PEDOT:PSS-based conductive fabric with distinctive properties for different application areas. The bending length analysis proved that the flexural rigidity was lower with higher PDMS-b-PEO to PEDOT:PSS ratio while tensile strength was increased. The SEM test showed that the smoothness of the fabric was better when PDMS-b-PEO is added compared to PEDOT:PSS alone. Fabrics with electrical resistance from 24.8 to 90.8 kΩ/sq have been obtained by varying the PDMS-b-PEO to PEDOT:PSS ratio. Moreover, the resistance increased with extension and washing. However, the change in surface resistance drops linearly at higher PDMS-b-PEO to PEDOT:PSS ratio. The conductive fabrics were used to construct textile-based strain, moisture and biopotential sensors depending upon their respective surface resistance.

## 1. Introduction

Poly(3,4-ethylenedioxythiophene) polystyrene sulfonate (PEDOT:PSS) conductive polymer is well-known for its high conductivity and applications in conductive synthetic textiles. It has been used with encouraging results as electrodes for flexible electronics. Unfortunately the use of PEDOT:PSS is currently constrained by its brittleness and limited processability. As a result many researchers have been trying preparing PEDOT:PSS-based conductive polymer composites. For instance, PEDOT:PSS and graphene oxide (GO) as an efficient alternative structure for indium tin oxide (ITO) in organic photovoltaics [1], ITO-PEDOT:PSS/poly(3-hexylthiophene):phenyl-C61-butyric acid methyl ester/Al [2], poly (vinyl alcohol) (PVA)–PEDOT:PSS blend filled with synthesized GO and reduced GO by solvent casting technique [3], PEDOT:PSS@polyurethane nonwovens by electrospinning and dip-coating [4], super paramagnetic PEDOT/magnetite nano particles [5], GO/glucose/PEDOT:PSS super capacitor [6], graphene and poly(3,4-ethylenedioxy thiophene)–poly(styrenesulfonate) (G-PEDOT:PSS) [7] an electroactive bacterium, Shewanellaoneidensis MR-1, inside a conductive three-dimensional PEDOT:PSS matrix [8], thermoelectric PEDOT:PSS with polyurethane [9], UV-ozone treated GO/PEDOT:PSS [10], graphenenano-platelets with PEDOT:PSS solutions to produce conductive, breathable, and light-weight mercerized cotton fabrics by spray coating [11]. PVA, phosphoric acid, PEDOT:PSS, and silver flakes [12], 3D graphene–PEDOT:PSS skeleton with poly(dimethylsiloxane) (PDMS) [13], and single-walled carbon nanotubes/PEDOT:PSS coated Tenano-rod composite films [14] have all been successfully developed. Though promising results have been found, a truly textile-based conductive device with adequate flexibility and stretchability without intensive impact on the bulk property of the textile fabric is still required.

On the other hand highly flexible and biocompatible conductive polymer composites are required. Because it is biocompatible, transparent, gas permeable, and economical, PDMS is widely used in medical research and technology, and there are a wide array of manufacturing techniques used for forming PDMS including soft-lithography and its derivatives, molding, dip casting, spin coating and many others. Besides, PDMS has a dielectric constant and a tunable elasticity which make it suitable for flexible electronic sensors [15]. As a result, PDMS-based flexible composites have been studied by many researchers. For example, high strain biocompatible PDMS-based conductive graphene and multi-walled carbon nanotube as a nano-composite strain sensors [16],electrically conductive PDMS-grafted carbon nanotubes-reinforced silicone elastomer [17], enhanced conductivity behavior of PDMS hybrid composites containing exfoliated graphite nano-platelets and carbon nano-tubes [18], high electro-conductive PDMS/short carbon fiber binary composites with electrical conductivity of 1.67 × 10^2^ S/m [19], conductive elastomers based on multi-walled carbon nanotubes in PDMS with up to 0.01 S/cm conductivity [20], silver nano-wire network embedded in PDMS as a stretchable, transparent, and conductive substrate with 15 Ohm/sq [20], and stretchable electronics based on Ag-PDMS PCB (Printed circuit board) with a typical resistance of 2 Ohms/cm [21], have been reported.

It was noticed that the common limitations of the conductive polymer composite-based conductive textiles reported in many works of the literature are that they possess inadequate flexibility, stretchability, and biocompatibility. Moreover, technical and scientific experimental evidence about the effect the conductive polymer composite has on the textile bulk properties like flexural rigidity, tensile strength, and extension at break, were not reported, which does not allow to determine if the fabrics still remain a true textile or if they lost their texture.

**Figure 1:**
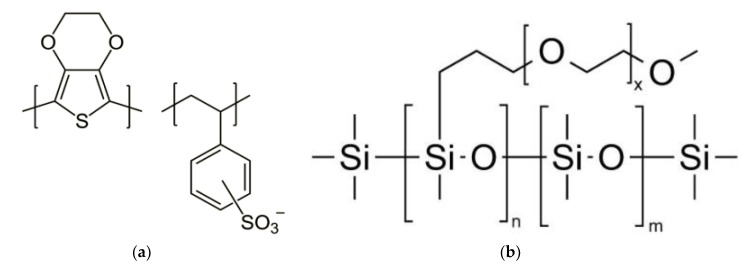
Chemical structure: (**a**) polystyrene sulfonate (PEDOT:PSS); (**b**) poly(dimethylsiloxane-b-ethylene oxide) (PDMS-b-PEO).

Therefore, another approach is introduced in this work to produce a PEDOT:PSS Clevios PH 1000 (Figure 1a) and PDMS-b-PEO (Figure 1b) conductive polymer composite-based fabric. As fabric, we selected knitted cotton, as cotton fabric is available everywhere and making cotton conductive will make access to conductive fabric easier. In addition, this work contains an in-depth study on the properties of the conductive textile, not only from an electronically point of view, but also as a textile material. The effect of the conductive polymer composite on bending length, flexural rigidity, tensile strength, and extension at break, thickness, add-on and weight has been studied.

## 2. Materials and Methods

### 2.1. Material and Chemicals

The conductive textile fabric produced consists of two parts: a water repellent textile substrate and an electro-conductive polymer composite. A weft knitted cotton fabric (S0) with 140 GSM and 0.5 mm thickness obtained from UGent, MaTCh laboratory was used as the textile fabric. Nano Spray water repellent (WR) for textile obtained from Lab@Home, Netherlands was utilized. PEDOT:PSS PH1000 Clevious conductive polymer obtained from Ossila Ltd. (Sheffield, UK) and poly(dimethylsiloxane-b-ethylene oxide) methyl terminated (PDMS-b-EO) obtained from Polyscience, Inc. (Warrington, UK) were used to produce the conductive polymer composite. The sheet resistance of each conductive polymer composite (PEDOT:PSS/PDMS-b-PEO) screen-printed fabric with different dimensions (sample sizes) was measured by a two point-method.

### 2.2. Methods

#### 2.1.1. Fabric Water Repellency Pre-Treatment

**Figure 2:**
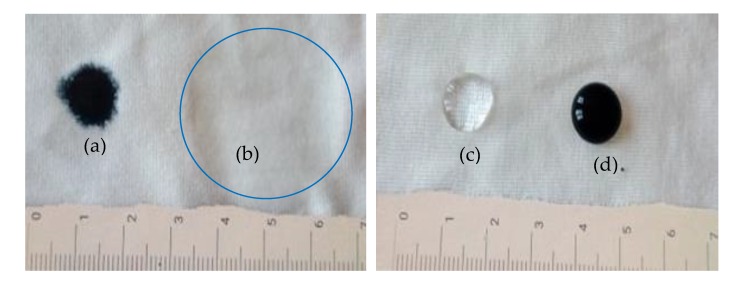
Effect of WR treatment on knitted cotton fabric: (**a**) PEDOT:PSS polymer dispersion on untreated fabric (S0); (**b**) drops of water on untreated fabric (S0); (**c**) drops of water on water repellent (WR)-treated fabric (S1); (**d**) PEDOT:PSS polymer dispersion on water repellent (WR)-treated fabric.

Before applying the PEDOT:PSS/PDMS-b-PEO conductive polymer composite (CPC), the fabric was pre-treated with WR to impart a hydrophobic effect on the fabric surface, and therefore prevent absorption of CPC into the fabric structure giving a confined CPC distribution within the required area. 3% owf (own weight of fabric) WR was gently sprayed on the fabric surface and then dried at 80 °C for 3 min. A drop test with water and PEDOT:PSS performed on the fabric showed an effective hydrophobicity as shown in Figure 2.

#### 2.2.2. Flat Screen Printing

Flat screen printing was preferred for this work because attaining the required conductive polymer composite paste viscosity and transferring it to the substrate by this technique is straightforward. Other possible techniques that could be explored are transfer printing [22], while also mechanics designs via transfer printing may provide another effective route toward stretchable conductive fabric [23].

**Figure 3:**
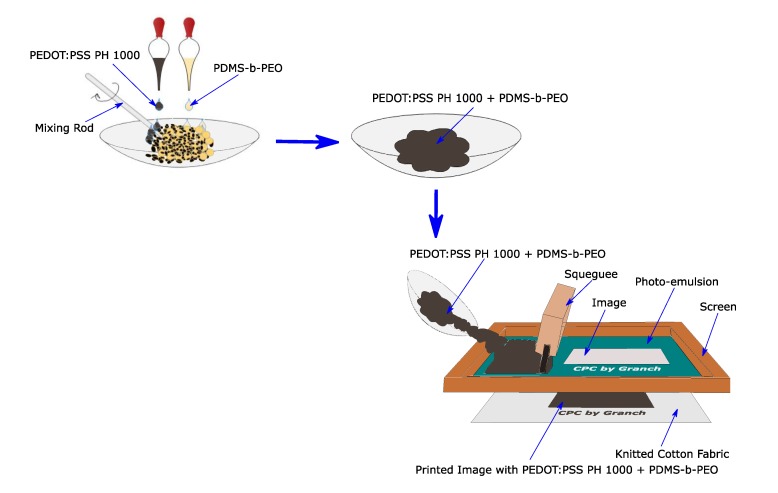
Screen printing of conductive polymer composite on knitted cotton fabric.

Different amounts of PDMS-PEO was manually mixed with a fixed amount of PEDOT:PSS (4 ml) for 3 min at room temperature. A mixing rod was used to prepare a homogenous blend of the two polymers. It was observed that stirring of the polymers showed a shearing property, the mixture solution was converted into a thicker paste which is convenient for screen printing. As a result, any thickening agent like present in conventional screen printing was not employed. Then the paste was applied to the surface of WR-treated cotton fabric (S1) via flat screen printing. A gentle agitation of the PEDOT:PSS and PDMS-b-PEO mix formed a thick paste and then the paste was simply swept over the flat screen mesh by a mini squeegee to improve the distribution. The overall printing process for this work is schematically represented in Figure 3.

**Figure 4:**
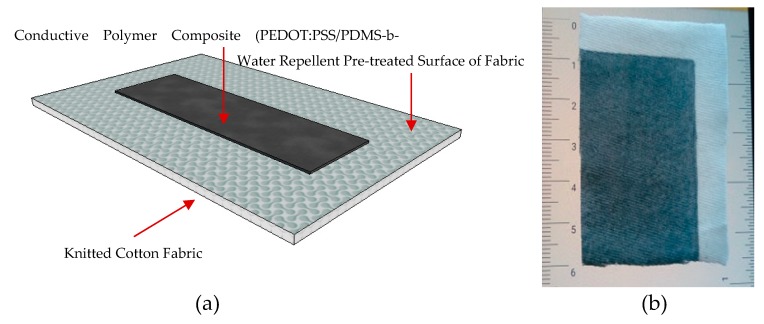
(**a**) Schematic design of the conductive textile fabric; (**b**) actual conductive textile fabric.

After the printing process, drying was performed in an oven at 70 °C for 5 min and cured at 150 °C for 5 min. The printed samples were thoroughly washed with distilled water. An example of the actual conductive knitted fabric produced is shown Figure 4.

To study the effect of PDMS-b-PEO on surface resistance, ten different ratios of PDMS-b-PEO to PEDOT:PSS (Table 1) were prepared and applied on the previously WR-treated knitted fabric via screen printing over an area of 62.5 cm^2^ (12.5 cm × 5 cm). The solid add-ons of the PEDOT:PSS/PDMS-b-PEO conductive polymer composite on the textile fabric were calculated using Equation (1).

w = (W_a_ − W_b_)/A, (1)

where, W_a_ = weight of fabric after printing in g and W_b_ = weight of fabric before printing in g, A = coated surface area in cm^2^ and w = solid add on per coated surface area (A) in g/cm^2^. 

The respective solid add-ons (w) for each percentage volume mix ratio of PDMS-b-PEO to PEDOT:PSS are provided in Table 1. The percentage volume mix ratio was calculated using Equation (2).

r = (V_e_/V_c_) × 100, (2)

where, V_e_ = volume of PDMS-b-PEO in ml, V_c_ = volume of PEDOT:PSS in ml, and r = percentage volume mix ratio of PDMS-b-PEO to PEDOT:PSS in %.

#### 2.2.3. Mechanical Characterization

Thickness: The thickness was determined using ISO 5084:1996(E) (determination of thickness of textile and textile products) using “Mitutoyo Digimatic Indicator.”

Bending Analysis: The bending length was measured according to the test method BS 3356:1990using a bending meter. Using the appropriate mean value, we calculated the flexural rigidity G, in mg cm, separately for the warp and weft directions by Equation (3).

G = 0.1MC^3^, (3)

where, G = flexural rigidity (mg cm), C = bending length (cm), M = mass/area of the specimen (g/m^2^).

Tensile Strength and Elongation at Break: The strength and elongation at break were tested using INSTRON universal strength tester. A tensile test according to ISO 13934-1 was used.

SEM analysis of CPC: The surface topology, cracks, holes, and appearance of yarns within the fabric before and after coatings were studied using FEI Quanta 200 FFE-SEM. Images were taken with an accelerating voltage of 20 kV. The non-conductive samples were prepared prior to analysis by applying a gold coating using Balzers Union SKD 030 sputter coater.

#### 2.2.4. Electrical Characterization

**Figure 5:**
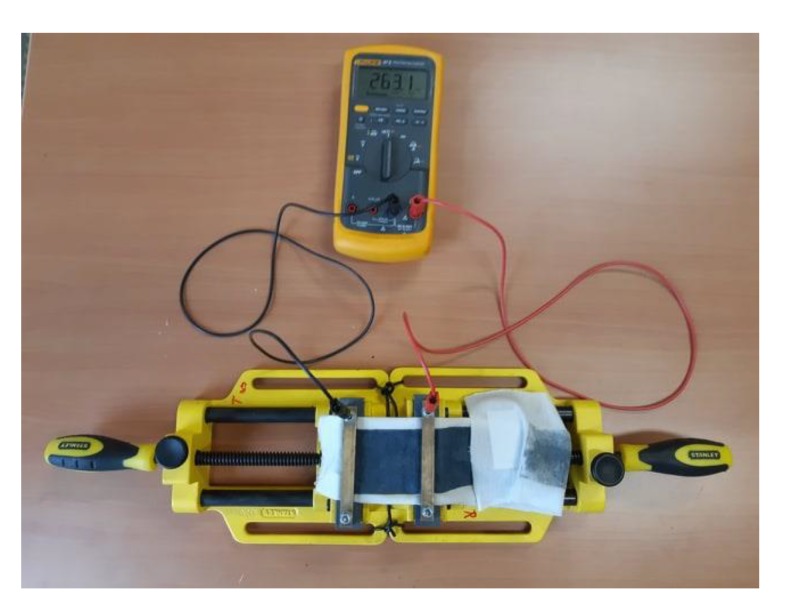
Two-point method resistance measurements set-up.

The sheet resistance of all samples was measured by using two-point methods. The samples were placed in a 3-7/8" MaxSteel Light Duty Drill Vise 83070 Stanley Hand Tool on both ends to make them stable for measurement as presented in Figure 5. The effect of stretching on sheet resistance has been studied from 0 to 35% elongation. The effect of repeated stretching on the sheet resistance has been studied by stretching the samples to their infliction point within five seconds, releasing from stretch and measuring the sheet resistance after 5 s.The stretching and releasing have been continued until the sheet resistance reached an infinite value.

In addition, we have constructed a 2.5 × 5 cm strain and moisture electrode from sample r60 as a demonstrator. We have also developed a PEDOT:PSS/PDMS-b-PEO (4:1) coated cotton fabric with a 332.5 Ω sheet resistance by increasing the add-on to 0.013 g/cm^2^ and tested this for electrocardiography (ECG) and electroencephalography (EEG) electrodes.

**Figure 6:**
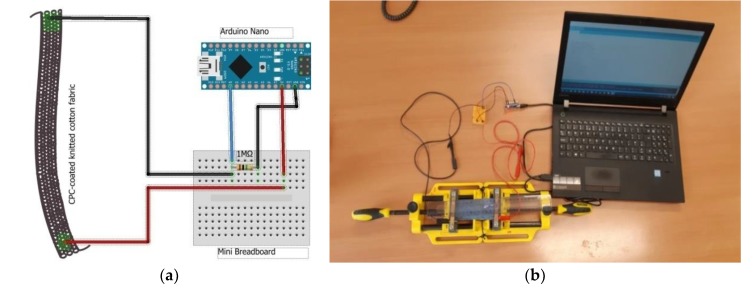
(**a**) Arduino Nano electronic circuitry design; (**b**) resistance measurements set-up.

To study the sensing stability of the conductive polymer composite-treated fabric an Arduino Nano set-up for this particular purpose with the circuitry in Figure 6 was used. One end of the PEDOT:PSS/PDMS-b-PEO CPC-treated fabric was connected to 5V of Arduino Nano input and the other was connected to 1MΩpull-down resistor and analog-to-digital converter (ADC) input. An IDE program suitable for reading out dynamically the resistance of the CPC was written. Then, the percentage change in resistance at its respective change in elongation from 0 to 35% was calculated using Equation (4).

∆R = 100 × (R_f_ − R_i_)/R_i_, (4)

where, ∆R = percentage change in resistance (%), R_i_ = initial resistance (kΩ), R_f_ = final resistance (kΩ).

In addition, the effect of washing on the surface resistance was measured. PEDOT:PSS/PDMS-b-PEO-coated knitted cotton fabric specimens of 4 × 4 cm were sandwiched between cotton fabric and polyester fabric and then sewn along all the four sides to form a composite specimen. Washing solution containing 5 g/l soap was taken into the launder-o-meter with a liquor ratio of 1:40. The specimen was treated for 30 min at 30 °C at a speed of 40 revolutions per minute. The specimen was removed and rinsed in cold water. The stitch was opened on three sides and dried in shadow. The surface resistance after washing was measured using a multi-meter and the two-point method. Finally, the percentage change in surface resistance was calculated using Equation (4).

## 3. Result and Discussion

### 3.1. Mechanical Property Analysis

#### 3.1.1. Solid Add-on

The solid add-on (w) of the conductive polymer composite on the knitted cotton fabric obviously increased with the increase in the amount of PDMS-b-PEO percentage mix ratio as represented in Table 2. This increase in add-on due to the increase in the amount of PDMS-b-PEO may raise the sheet resistance as the conductive component could probably be trapped inside the PDMS-b-PEO elastomer.

#### 3.1.2. Thickness Analysis

**Figure 7:**
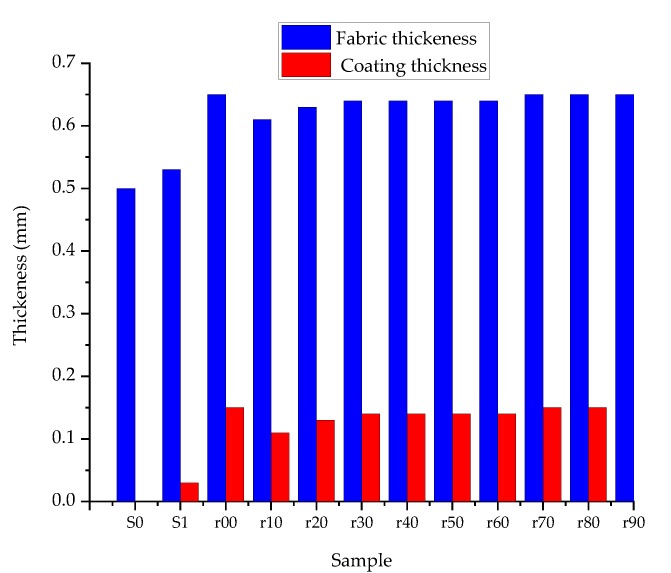
Effect of PEDOT:PSS/PDMS-bb-PEO on thickness.

From Figure 7, the thickness of the fabric increased by 0.03 mm when WR is applied. After the conductive polymer composite has been applied, the thickness has moreover increased by less than or equal to 0.14 mm until a percentage mix ratio of 60% and continues with a thickness equal to the sample r00 until the mix ratio is 90% (sample r90). This shows that the presence of PDMS-b-PEO has less effect on the thickness than when PEDOT:PSS alone (sample r00) was utilized. This could have a positive contribution to the flexibility of the fabric.

#### 3.1.3. Bending Length Analysis

In Table 3, we presented the measured values of bending length and respective calculated flexural rigidity according to Equation (3). The fabric became stiffer when treated with a water-repelling agent, as evidenced by an increase in bending length of 29%. The fabric became again stiffer when the CPC was applied, with double the bending length of the r00 sample over the S0 sample (base cotton fabric). Moreover, this increase in bending length was more intensive when PEDOT:PSS alone was used. For instance, the bending length of the PEDOT:PSS-treated fabric decreased on an average by 11% when r was 50% (sample r50) and the flexural rigidity reduced by 13%.

#### 3.1.4. Tensile Strength

**Figure 8:**
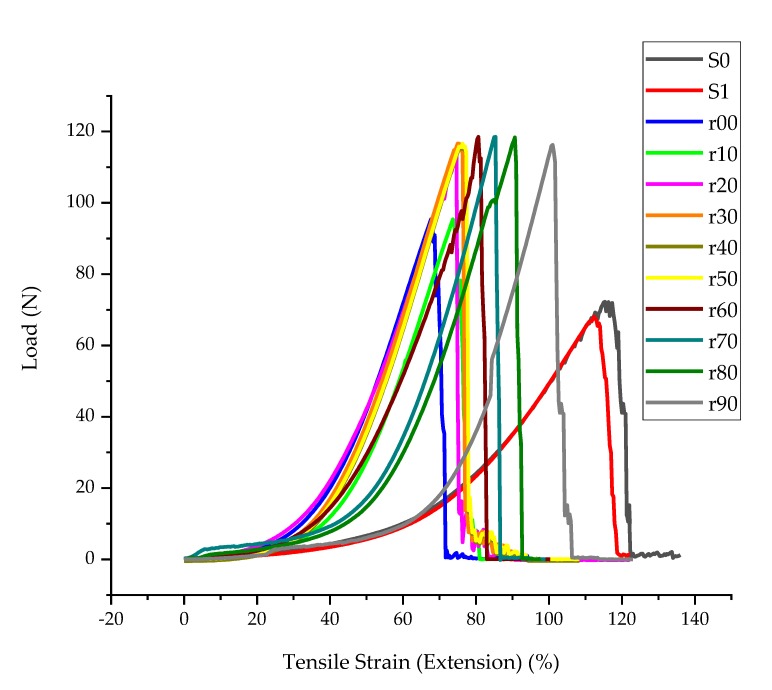
Load-elongation curve.

From the load-elongation curve in Figure 8, both the tensile strength and strain at break of the fabric reduced from 72.2 to 68.1 N and 115.3 to 112.3% because of the water repellent treatment. This could be due to the effect of the water repellent that might cause a very slight degradation during drying and curing, but is overall a minor influence on the textile properties.

The application of only PEDOT:PSS (r00) increased the tensile strength in line with a stiffer fabric as found in the bending testing, but the strain was greatly decreased. This indicates that the PEDOT:PSS on the surface adds to the strength of the fabric making it stiffer, but reduces the stretch. The addition of PDMS-PEO to the fabric gives again higher tensile strength and allows again higher strain. Further increase of PDMS-PEO ratio has little influence on the strength, but does further increase the strain. For instance, a shift of tensile strength from 78.2 to 115.8 N and a strain 69.7 to 77 % at break was observed when r shifts from 0% to 50%. In general, the tensile strength becomes constant at around 118 N. Therefore, it is rational to say the new conductive polymer composite gives smaller Young’s modulus which is important for the e-textile application.

#### 3.1.5. SEM Characteristics of the Conductive Polymer Composite

**Figure 9:**
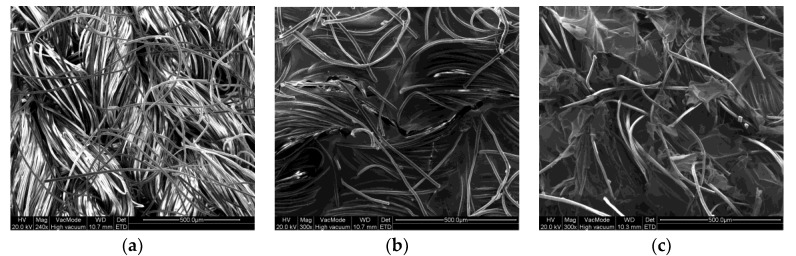
SEM: (**a**) WR treated; (**b**) PEDOT:PSS coated; (**c**) PEDOT:PSS-PDMS:PEO coated.

The SEM results (Figure 9) showed that the yarn loop interstices in the fabric were coved by the addition of PEDOT:PSS/PDMS-b-PEO conductive polymer composite. Protruding loops were also observed in the base fabric but not after the printing. As a result, the coated fabrics are smoother than uncoated fabrics. The presence of PDMS-PEO further improved the smoothness and the coverage of the yarn loop interstices. The surface of the PEDOT:PSS-treated fabric looks more glassy when PDMS-b-PEO was added.

### 3.2. Electrical Characteristics Analysis

#### 3.2.1. Effect of PDMS-b-PEO to PEDOT:PSS Ratio on Resistance

**Figure 10:**
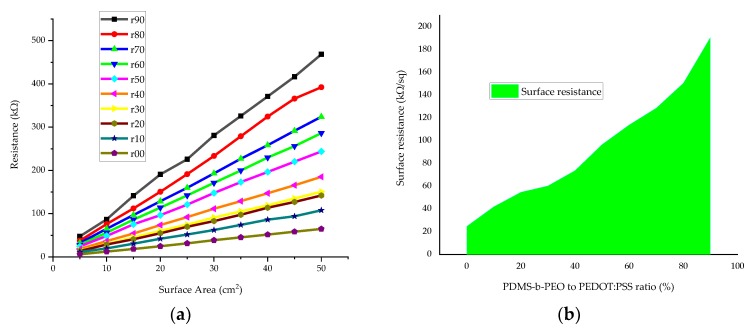
Effect of PDMS-b-PEO concentration on resistance: (**a**) effect of PEDOT:PSS to PDMS-b-PEO ratio on resistance at different surface area, width kept constant; (**b**) effect of PDMS-b-PEO on surface resistance.

For all the samples, the resistance increases with increase in the surface area when the length was increased by keeping the width constant. Therefore, it is obvious that resistance drops with increasing concentration of the non-conductive polymer and the surface area. The effect of the PDMS-b-PEO to PEDOT:PSS ratio on the surface resistance is shown in Figure 10.

In all the samples, it was observed that the reproducibility was fairly good but still needs improvement. This could arise from the homogeneity of the polymer blend, uneven distribution of the conductive polymer composite during screen printing, and the uneven property of the textile fabric at its different portions.

**Figure 11:**
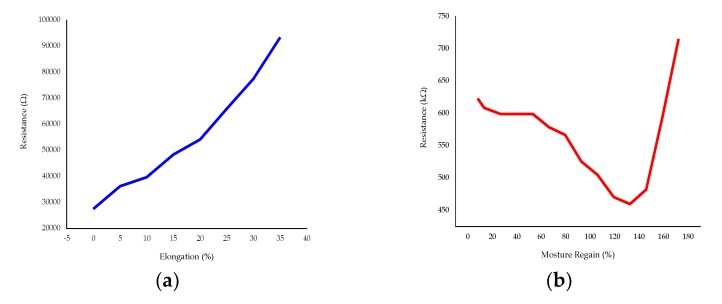

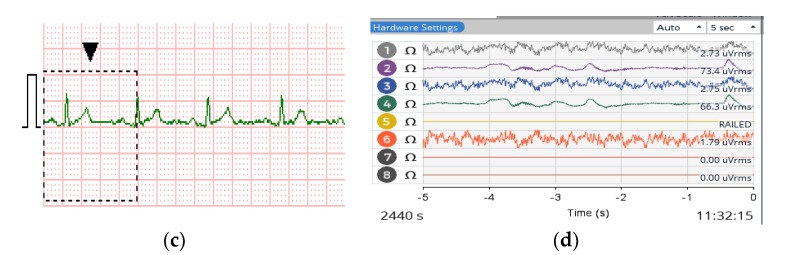
PEDOT:PSS/PDMS-b-PEO coated cotton electrodes for: (**a**) strain dynamic response at 0.67 cm/s rate of stretching for six seconds; (**b**) moisture dynamic response at1 ml/s rate of water spray for six seconds; (**c**) electrocardiography (ECG) signal using PC-80B; (**d**) electroencephalography (EEG) signal using OpenBCI board.

As the obtained resistance covers a big range from 6 to 468.4 kΩ (Figure 10b) depending on the concentration of PDMS-b-PEO and the surface area of the treated fabric, they can be used as a textile-based electrode for different sensors such as strain, moisture, biopotential (EEG, ECG), interconnection, energy storage, and other applications. For instance, in the strain and moisture sensor electrode demonstration of sample r60, it showed a linear increase in resistance during the stretch to its infliction point. The resistance also dropped up to 132.5% moisture regain, while above 132.5%, the resistance rapidly increased, which may be due to swelling of the PEDOT:PSS at higher moisture regain. Moreover, the ECG and EEG electrodes constructed from the PEDOT:PSS/PDMS-b-PEO (4:1) coated cotton fabric (332.5 Ω) at an add-on of 0.013g/cm^2^, showed good qualities of collected ECG and EEG waveforms. The strain, moisture, ECG and EEG responses from the PEDOT:PSS/PDMS-b-PEO-coated cotton fabric electrodes are shown in Figure 11.

The surface resistance increased from 24.8 kΩ/sq to 96.7 kΩ/sq as the PDMS-b-PEO to PEDOT:PSS ratio increases from 0 to 90% (Figure 10b). Thus, the ratio determines the application area, as each requires specific surface resistance value. When high resistance is required, one can select a higher concentration of PEDOT-b-PEO and when lower resistance is required a lower ratio. Instead of the high resistance values obtained in this paper, one can also obtain lower resistance as mentioned in the case of an add-on of 0.013 g/cm^2^, where we obtained 300 Ω/sq on the same fabric.

#### 3.2.2. Effect of Stretching on Sensing Stability

**Figure 12:**
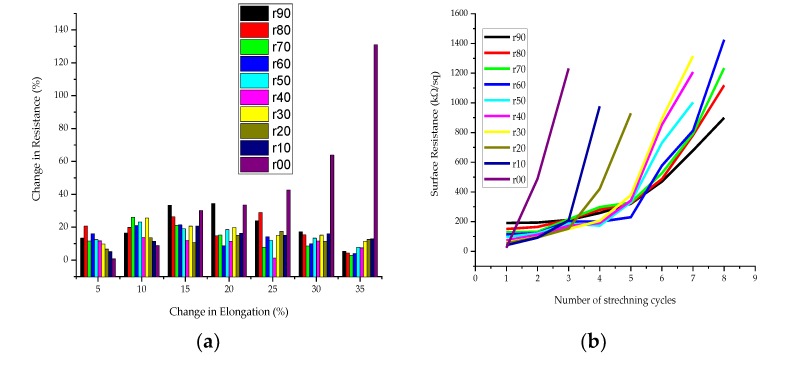
Effect of stretching on surface resistance and sensing stability: (**a**) Change in resistance due to stretching; (**b**) surface resistance at different stretching cycles.

Figure 12a shows that the stretching of the PEDOT:PSS/PDMS-b-PEO has a complex effect on the surface resistance in all samples for up to 35% elongation. In all cases, the resistance increased over the 0% elongation. This could be due to a decrease in the density of conductive components during stretch. It was observed that the change in resistance due to stretching increases at the beginning of stretching and then decreases with increasing amount of PDMS-b-PEO, with the inflection point depending on the ratio r. Whereas, when r is zero (sample r00), the percentage change in resistance increases with stretching. Moreover, the change in resistance is smaller when PDMS-b-PEO was employed. For instance, the change in resistance of sampler00 was 0.74% which is smaller than sample r60 i.e., 16% when the change in elongation was 5%. But, when the change in elongation reached 35%, the change in resistance of r60 i.e., 4% was smaller than that of r00 i.e., 131%. In addition, increasing the ratio of PDMS-b-PEO to PEDOT:PSS showed a better surface resistance recovery after stretching and better stability when re-stretched up to eight cycles. The surface resistance of sample r00 reached an infinite value at three cycles. Whereas, the most stable sample, sample r90, stayed conductive up to eight stretching cycles to its infliction point. The surface resistance of the samples after recovering from each stretch and 5 s rest is shown in Figure 12b. Though the sensing stability is not bad, it needs further improvement. A specific sample with PDMS-b-PEO can only be used to sense stretch up to the infliction point of that sample, as afterward the strain to resistivity change is unstable. Further refinement in the way of making the composite or coating technique may solve this problem.

#### 3.2.3. Effect of Washing on Surface Resistance

**Figure 13:**
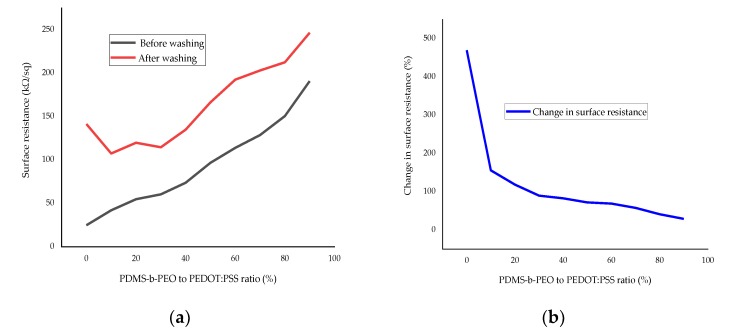
Effect of washing on the surface resistance at different PDMS-b-PEO to PEDOT:PSS ratios: (**a**) surface resistance before and after washing; (**b**) percentage change in surface resistance due to washing.

Figure 13 shows that the resistance increases after a single wash. But, the increase in surface resistance due to washing decreased from ~470% to ~30% with increasing r ratio from 0 to 90. Thus, the washing fastness improved with the increase in the amount of PDMS-b-PEO. This property of the fabric with CPC should be improved further if it is going to be used for frequently washed products.

## 4. Conclusions

In this work we have successfully developed a stretchy and flex conductive textile fabric with improved sensing stability of stretch and better washing fastness via screen printing of PEDOT:PSS and PDMS-b-PEO polymers. It was observed that the increase in the proportion of PDMS-b-PEO increases the tensile strength at break. The bending length analysis proved that the flexural rigidity drops at higher PDMS-b-PEO to PEDOT:PSS ratio which shows as the PDMS-b-PEO imparts flexibility over the pure PEDOT:PSS sample. SEM results showed that the presence of the conductive polymer composite brought smoothness and better coverage of yarn loops interstices. Samples with PEDOT:PSS/PDMS-b-PEO conductive polymer composite showed less protruding yarn loops than PEDOT:PSS alone. We found different electrical characteristics that range from 24.8 to 190.8 kΩ/sq before washing when the ratio of PDMS-b-PEO to PEDOT:PSS varies from 0 to 90%. The conductive character remained after washing, but was decreased, though much less so if PDMS-b-PEO is present. Moreover, the presence of PDMS-b-PEO improved the surface resistance recovery after release from stretching and remained conductive for more numbers of stretching cycles than sample r00.We have also realized that the surface resistance can be dropped further by increasing the add-on of the conductive polymer composite. These different electrical resistance characteristics could be chosen based on the application required to use them for sensors, interconnections, antenna, storages, and others. For instance, the conductive fabrics with higher resistance can be used to develop a textile-based humidity sensor, the middle ones can be used for the strain sensor and the lower ones can be for dry ECG, EMG, and EEG electrodes. A deep study on the electrical and physical characteristics of the developed samples is necessary to exactly indicate the correlation between the produced samples and end-use.

## Figures and Tables

**Table 1:** Mix ratios of the conductive polymer composite and weight before printing.

**Sample**	**r ** **[%]**	**V_c_** ** [ml]**	**V_e_** ** [ml]**	**W_b_** ** [g]**	**A** ** [cm^2^]**
r00	0	4	0	1.655	5 × 12.5
r10	10	4	0.4	1.653	5 × 12.5
r20	20	4	0.8	1.629	5 × 12.5
r30	30	4	1.2	1.608	5 × 12.5
r40	40	4	1.6	1.578	5 × 12.5
r50	50	4	2	1.645	5 × 12.5
r60	60	4	2.4	1.678	5 × 12.5
r70	70	4	2.8	1.661	5 × 12.5
r80	80	4	3.2	1.622	5 × 12.5
r90	90	4	3.6	1.628	5 × 12.5

**Table 2:** Solid add-on of coated samples.

**Sample**	**V_c_** ** [ml]**	**V_e_** ** [ml]**	**W_b_** ** [g]**	**W_a_** **[g]**	**A ** **[cm^2^]**	**w ** ** [g/cm^2^]**
r00	4	0	1.655	1.913	5 × 12.5	0.0041
r10	4	0.4	1.653	2.142	5 × 12.5	0.0078
r20	4	0.8	1.629	2.344	5 × 12.5	0.0114
r30	4	1.2	1.608	2.401	5 × 12.5	0.0127
r40	4	1.6	1.578	2.462	5 × 12.5	0.0141
r50	4	2	1.645	2.44	5 × 12.5	0.0144
r60	4	2.4	1.678	2.646	5 × 12.5	0.0155
r70	4	2.8	1.661	2.671	5 × 12.5	0.0162
r80	4	3.2	1.622	2.688	5 × 12.5	0.0171
r90	4	3.6	1.628	2.733	5 × 12.5	0.0177

**Table 3:** Bending length and flexural rigidly results.

Fabric	Weight (g/m^2^)	Bending Length (cm)					Flexural Rigidity (mg cm)				
		Wale Direction		Course Direction		Average	Wale Direction		Course Direction		Average
		Face	Back	Face	Back		Face	Back	Face	Back	
**S0**	140.0	1.3	1.2	1.3	1.7	1.4	27.3	24.2	27.3	71.2	34.8
**S1**	146.0	1.8	1.7	1.7	1.9	1.8	78.2	65.6	71.7	100.1	78.2
**r00**	176.0	3.0	2.9	2.9	2.5	2.8	475.2	407.4	407.4	262.0	382.2
**r10**	191.0	2.9	2.8	2.8	2.4	2.7	456.3	397.2	428.3	247.9	375.9
**r20**	199.0	2.9	2.7	2.7	2.3	2.7	460.7	396.1	409.4	245.3	371.4
**r30**	205.0	2.8	2.7	2.7	2.2	2.6	454.9	394.6	408.0	230.4	364.5
**r40**	209.0	2.8	2.7	2.6	2.2	2.6	453.9	388.9	371.6	219.5	350.6
**r50**	213.0	2.7	2.6	2.6	2.1	2.5	433.4	383.1	353.2	200.1	333.8
**r60**	216.0	2.7	2.6	2.5	2.1	2.5	415.8	370.9	341.6	191.6	321.6
**r70**	221.0	2.6	2.5	2.5	2.0	2.4	392.9	349.5	341.2	179.5	307.4
**r80**	224.0	2.5	2.5	2.5	1.9	2.3	362.8	337.6	333.5	153.6	287.0
**r90**	228.0	2.5	2.5	2.4	1.9	2.3	356.3	335.3	315.2	149.1	279.2
